# Diverse Methods with Stereoselective Induction in the Asymmetric Biginelli Reaction

**DOI:** 10.3390/molecules29163864

**Published:** 2024-08-15

**Authors:** Marcos Díaz-Fernández, Manuel Algarra, Saturnino Calvo-Losada, José-Joaquín Quirante, Francisco Sarabia, María-Soledad Pino-González

**Affiliations:** 1 Department of Organic Chemistry, Faculty of Sciences, University of Málaga, 29071 Málaga, Spain; marcos_diaz_fernandez@uma.es (M.D.-F.); frsarabia@uma.es (F.S.); 2 Department of Physical Chemistry, Faculty of Sciences, University of Málaga, 29071 Málaga, Spain; asenruhup@hotmail.com (S.C.-L.); quirante@uma.es (J.-J.Q.); 3 Department of Science, INAMAT2-Institute for Advanced Materials and Mathematics, Public University of Navarra, 31006 Pamplona, Spain; manuel.algarra@unavarra.es

**Keywords:** pyrimidines, multicomponent reactions, asymmetric, enantioselective, N-heterocycles, Biginelli reaction

## Abstract

The relevance of the asymmetric Biginelli reaction (ABR) has been increased in this century, due to the pharmacological application of its products. This review focuses predominantly on articles published in the period from 2015 to 2024 on asymmetric synthetic advances in the formation of dihydropyrimidinones (DHPMs), dihydropyrimidinethiones (DHPMTs), and related compounds. The relevant bibliography on general processes in the Biginelli reaction and some methods of separation of isomers have also been referenced.

## 1. Introduction

Among the multicomponent reactions (MCRs) [1], one of the most studied since its discovery in 1891 is the Biginelli reaction [2,3], which produces dihydropyrimidines (DHPMs) as the main application. A large number of procedures have been published varying the type of starting compounds, catalysts, and solvents, emphasizing the variety of applications of their products [4]. Most of the procedures emphasized the importance of obtaining good reaction yields and optimal conditions. To attain these goals, the mechanism of the Biginelli reaction has been considered in several publications [5,6]. Given the pharmacological importance of Biginelli products [7,8], structure–activity correlation studies have been reported, but in a large number of cases, the studies have been carried out on racemic mixtures [9,10]. Recently, considering that in some structures one of the enantiomers might have a high biological activity and not the other (or even the opposite activity), the importance of obtaining enantiomerically pure DHPMs has been highlighted [11,12]. As a consequence, obtaining enantiomerically pure DHPMs has become a synthetic priority. Another possibility that has been explored is the separation of both enantiomers from their racemic mixtures. With that purpose, Kappe et al. performed chiral enantiomer resolution via the fractional crystallization of diastereomeric salts [13]. Blasco et al. reported a biocatalytic highly enantioselective synthesis of (*S*)-monastrol via enzymatic resolution. These authors utilized lipase enzyme to afford the (*R*) enantiomer in 48% yield (66% *ee*) and (*S*) in 31% yield (97% *ee*) [14]. More recently, a redox deracemization of 3,4-dihydropyrimidin-2-ones with phosphonate substituents at the C4-position has been performed, obtaining optically active DHPMs derivatives [15]. In another work, racemic ligands were separated by semipreparative HPLC on a chiral stationary phase. The obtained DHPMs were studied as antagonists of the human A_2B_ adenosine receptor [16]. The studies were developed considering structure–activity relationship and enantioselective recognition. In some examples, the (*S*) stereoisomer was shown to be almost twice as potent than the racemic mixture. Other published methods were based on the asymmetric reduction of 2-hydroxypyrimidines and H-substituted pyrimidin-2-ones [17,18]. In addition, with the aim of obtaining information on the individual capabilities of an enantiomer (in their application as potential pharmaceuticals), theoretical calculations have been carried out on specific DHPMs. Nonetheless, a non-asymmetric Biginelli route was utilized [19].

In addition to the Biginelli reaction, other methods have been published to stereoselectively obtain DHPMs, such as those involving cycloaddition reactions. Thus, in a review published by our group on this type of reaction, in the synthesis of DHPMs and related compounds, some stereoselective reactions of DHPMS have been described [20].

Chronologically speaking, the publication of studies on Biginelli asymmetric synthesis started off at the turn of the century. Thus, we can consider the pioneering work of Juaristi and Muñoz-Muñiz in 2003, using a chiral amide with CeCl_3_ and InCl_3_. However, only up to 40% *ee* was obtained [21]. Subsequently, Zhu and co-workers, in 2005 [22], described a more efficient enantioselective Biginelli reaction, reaching an *ee* superior to 99%, using a new chiral ytterbium catalyst. Later on, Gong’s research group reported the use of 1,1’-bi-2-naphthol (BINOL)-derived chiral phosphoric acids as organocatalysts, furnishing the DHPMs with up to 97% *ee* [23] Subsequently, in 2009, this group found out that the stereochemistry of the reaction could be reversed by adjusting the size of the 3,3′-disubstituents of the binaphthol phosphoric acids [24].

In this review, we will focus on the asymmetric Biginelli reactions that allow us to directly obtain the enantiomerically pure products. The publications carried out from 2015 to the beginning of 2024 will be preferentially taken into account. In the last few years, other review papers with information on ABRs have been published [1,4,12]. Even during the process of finalizing this manuscript, a new review has been reported [25] highlighting the current importance of ABRs in organic synthesis.

## 2. Strategies in the Asymmetric Biginelli Reactions

With the aim of obtaining enantiopure DHPs, several strategies have been carried out with the use of different types of catalysts.

### 2.1. Synthetic Chiral Organic Catalysts

In recent years, the Biginelli asymmetric reaction with synthetic chiral organic catalysts has been the most relevant method to obtain enantiopure DHPMs [12,25].

#### 2.1.1. Chiral Brønsted Acids

##### Phosphoric Acids

**Scheme 1:**
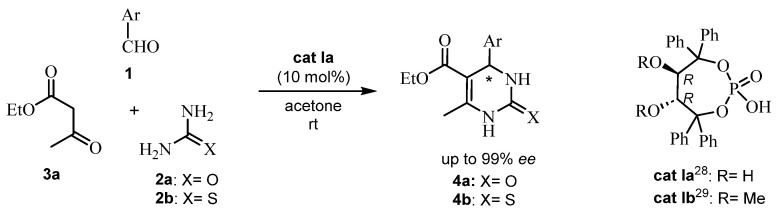
Asymmetric Biginelli reaction with **cat Ia**.

In the last twenty years, apart from ABRs, there has been a profusion of methods with chiral phosphoric acid for catalyzed asymmetric multicomponent reactions [26]. Surprisingly, highly enantioselective reactions could be observed by using non-optically pure phosphoric acids [27]. A derivative of L-tartaric acid was the choice by Hu et al. [28] to design a novel sterically hindered chiral cyclic phosphoric acid **cat Ia**. Its synthesis was based on a highly regioselective protection of hydroxyls 1 and 4 of chiral (*R*,*R*) 1,1,4,4-tetraphenylbutanetetraol. Good enantioselectivities were obtained by this asymmetric Biginelli reaction (up to 99% *ee*, see Scheme 1) [28].

**Scheme 2:**
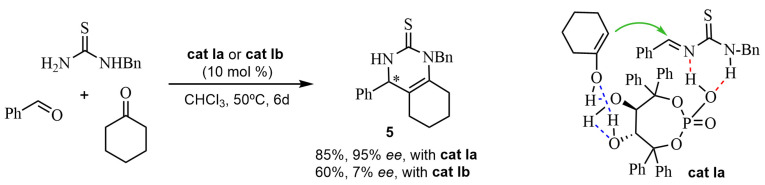
Comparative results with **cat Ia** or **cat Ib** and proposed chiral transition-state structure with **cat Ia**.

Lately (in 2020, [29]), the latter research group proposed a plausible chiral transition-state structure in the Biginelli-like reaction catalyzed by the phosphoric acid **cat Ia**, with cyclohexanone as an enolizable compound, to obtain the bicyclic asymmetric pyrimidines **5** (Scheme 2). In this model, the chiral **cat Ia** activates the intermediate imine. The two hydroxyl groups form intramolecular and intermolecular H-bonds with the enolizable ketone, favoring the stereoselective attack of the enol on the imine. When the methoxy derivative **cat Ib** was used as a catalyst, the enantiomeric excess was lower, indicating that the two hydroxyl groups were indispensable for attaining a higher enantioselectivity (Scheme 2).

**Scheme 3:**
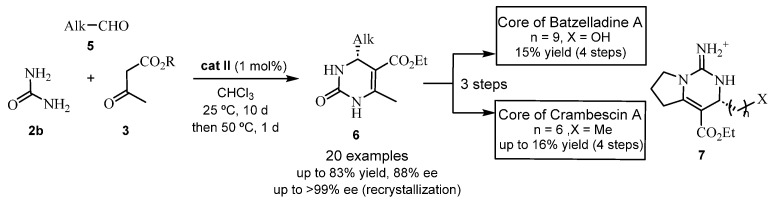
Asymmetric Biginelli reaction with **cat II**.

**Figure 1:**
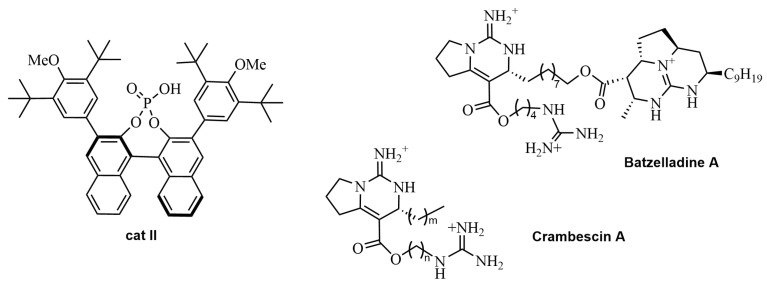
Structures of cat II, Crambescin A, and Batzelladine A.

Aliphatic aldehydes have also been catalyzed by chiral phosphoric acids in an ABR. Precisely, Guo et al. designed a synthetic strategy to obtain the bicyclic guanidine core **7** of Crambescin A and Batzelladine A in the presence of the catalyst **cat II** (see Scheme 3 and Figure 1) [30,31]. Thus, Crambescin A could be synthesized according to previously reported literature procedures [32]. Lately, a synthesis of (+)-Crambescin A was reported by Guo’s group, but without employing the Biginelli reaction [33].

**Scheme 4:**
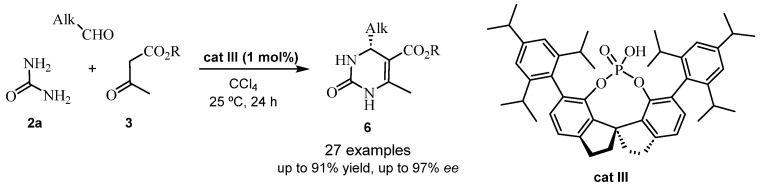
Asymmetric Biginelli reaction with **cat III**.

**Scheme 5:**
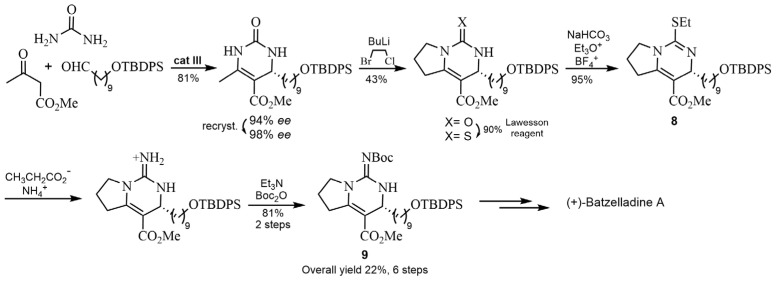
Synthesis formal of (+)-Batzelladine A by Biginelli reaction with **cat III**.

The spirocyclic phosphoric acid **cat III** was used in another asymmetric reaction to obtain 4-alkyl-3,4-dihydropyrimidin-2-(1*H*)-one (4-alkyl-DHPM) scaffolds with good yields and enantioselectivity (Scheme 4). Subsequent studies showed that this strategy was optimal for the synthesis of the structural core of Batzelladine A. Thus, this procedure [34] improved the results reported in the literature [32], providing the bicyclic guanidine fragment of (+)-Batzelladine A **9** in only 6 steps and 22% of total yield (compared to previous 12 steps and 13% of total yield, see Scheme 5) [32].

**Scheme 6:**
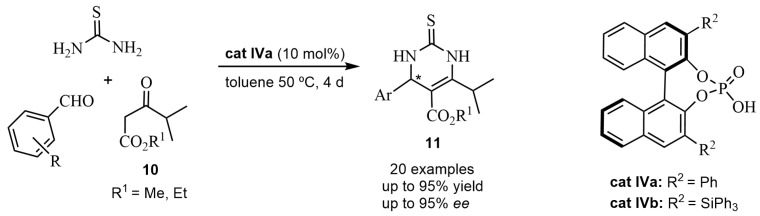
Asymmetric Biginelli reactions with **cat IV**.

On the other hand, taking into account that the chiral 6-isopropyldihydropyrimidines **11** are very important intermediates for statin drugs, an organocatalyzed ABR was designed [35]. The chiral BINOL-derived phosphoric acid **cat IVa** catalyzed the condensation of aromatic aldehydes, β-dicarbonyl compounds **10**, and thiourea, in the asymmetric synthesis of 6-isopropyl-3,4-dihydropyrimidines **11** obtained with good yields and enantioselectivities (Scheme 6). Recently, Chetty et al. in 2024 [36], studying the optimal solvent conditions for obtaining **11,** carried out the enantioselective reaction of nitrobenzaldehyde, acetoacetate, and thiourea in the presence of silylated **cat IVb**, (Scheme 6). It was shown that both toluene and cyclopentyl methyl ether gave equivalent results in both cases (99% yield and 92% *ee*).

**Scheme 7:**
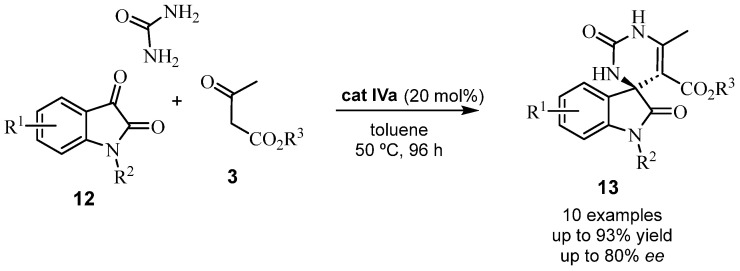
Synthesis of spiro(indoline-pyrimidine) dione derivatives **13**.

The ability of isatin to form part of various anticancer pharmacophores has been recently reviewed, highlighting its effectiveness in Biginelli-like reactions [37]. As a matter of fact, isatins act as carbonyl substrates in this type of reaction. Thus, an asymmetric Brønsted acid-catalyzed Biginelli-like reaction was reported, employing the *N*-substituted isatins **12**, urea, and alkyl acetoacetates. The BINOL-derived phosphoric acid **cat IVa** catalyzed the enantioselective formation of a small library of chiral, spiro(indoline-pyrimidine) dione derivatives **13** (Scheme 7). The absolute configuration of the new spiro stereocenter was assigned based on NMR studies of diastereomeric derivatives [38].

Other series of spirooxindole-dihydropyrimidinones and -dihydropyrimidinethiones with analogous structures to that of **13** were described by Maddela et al. who utilized Fe_3_O_4_ nanoparticles as a catalyst [39].

##### Sulfonic Acids and Derivatives

**Scheme 8:**
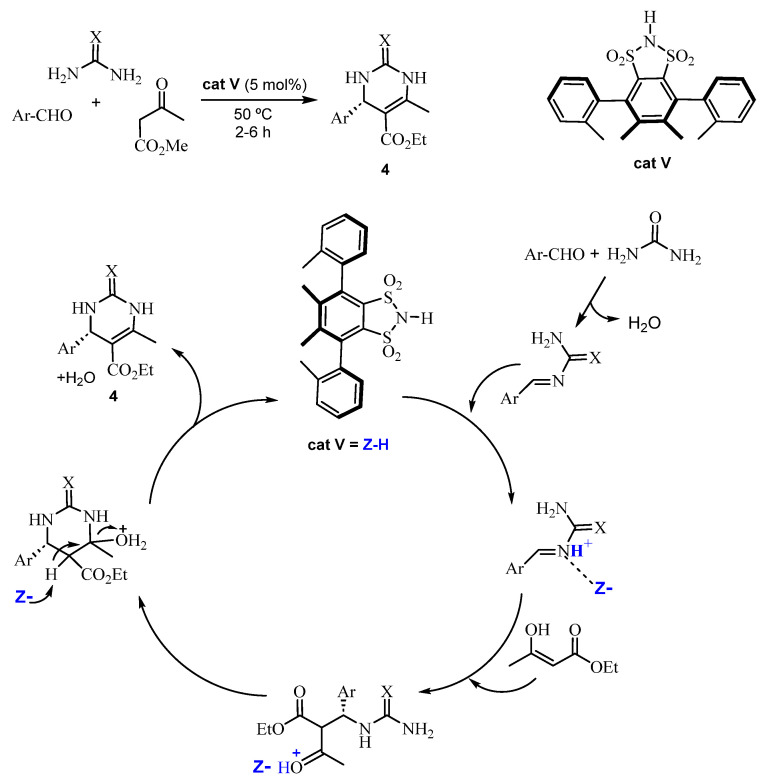
Synthesis and proposed mechanism for the enantioselective formation of DHPMs **4**.

Barbero et al. [40] reported the use of a derivative of 1,2-benzenedisulfonimide as a chiral Brønsted catalyst (**cat V**) for ABRs. These authors used different reagents (aromatic aldehydes, urea or thiourea and ethyl acetoacetate) and 5 mol% of the catalyst **cat V** under neat conditions and heating at 50 °C. High yields of DHPMs (average 91%) and enantiomeric excesses (average 97%) were achieved. In addition, they proposed a mechanism for the enantioselective formation of the DHPMs **4** with **cat V** (Scheme 8).

**Figure 2:**
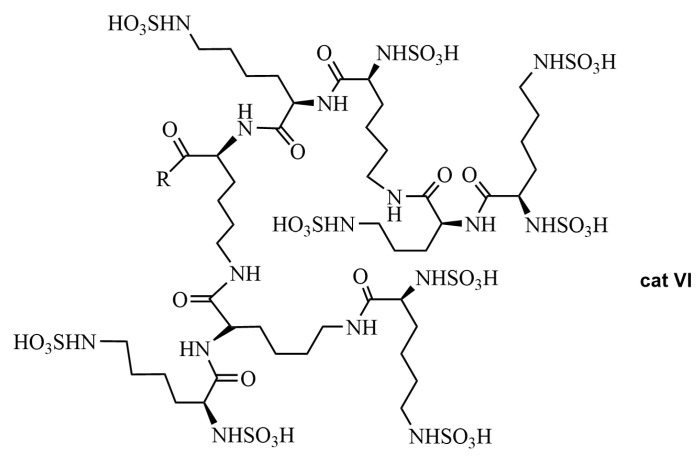
Sulfonic-functionalized hyperbranched polylysine (HBPL-SO_3_H) (**cat VI**).

**Scheme 9:**
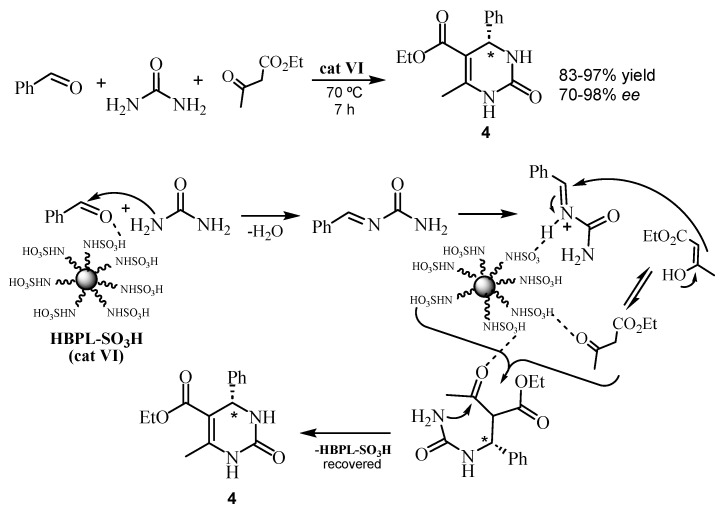
The proposed mechanism for the Biginelli reaction in the presence of the catalyst HBPL-SO_3_H (**cat VI**).

In 2023, Nasery et al. [41] reported a sulfonic-functionalized hyperbranched polylysine (HBPL-SO_3_H) (**cat VI**) as a novel and efficient organocatalyst in an asymmetric Biginelli reaction (Figure 2). All of the reactions were carried out with 1 mmol of aryl aldehyde, urea or thiourea, ethyl or methyl acetoacetate, and 0.03 g of HBPL-SO_3_H, under solvent-free conditions (70 °C, 7 h). The chiral dihydropyrimidinones **14** were obtained in 83–97% yield and 70–98% *ee* (determined by chiral HPLC analysis) (Scheme 9). HBPL-SO_3_H was synthesized in 98% yield, its structure was confirmed by IR, NMR, XRD, and TGA analyses, and it could be reused.

#### 2.1.2. Ionic Liquids with Chiral Organic Framework

The relevance of the role of ionic liquids (ILs) in the Biginelli reaction, alone or in combination with homogeneous and heterogeneous catalysts, is well established [42]. In the last few years, chiral ionic liquid catalysts have also been studied in relation to their role in asymmetric reactions [42].

**Scheme 10:**
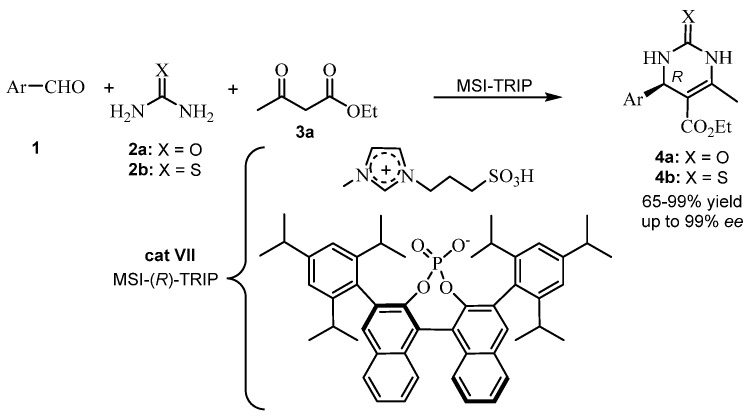
Combined catalyst MSI-(*R*)-TRIP (cat **VII**) in an ABR.

In 2018, Alvim et al. [43] studied the ionic liquid effect (ILE) combined with an asymmetric counteranion-directed catalysis (ACDC) in an ABR. Ionic liquids bearing chiral anions were chosen as catalysts for enantioselective Biginelli reactions (Scheme 10). The reaction mechanism (via iminium) was elucidated using electrospray (tandem) mass spectrometry (ESI-MS/MS). Chiral induction arises from a supramolecular aggregate where the anion and the cation of the catalyst [MSI-(*R*)-TRIP] (**cat VII**) are alongside a key cationic intermediate of the reaction, with each component being essential for the chiral induction success. The resulting enantiomeric excesses of the THPMs **14** were measured by chiral HPLC analyses. The absolute stereochemistry of one THPM was confirmed with the literature data, and only the (*R*) isomer was formed (Scheme 10) [43].

**Scheme 11:**
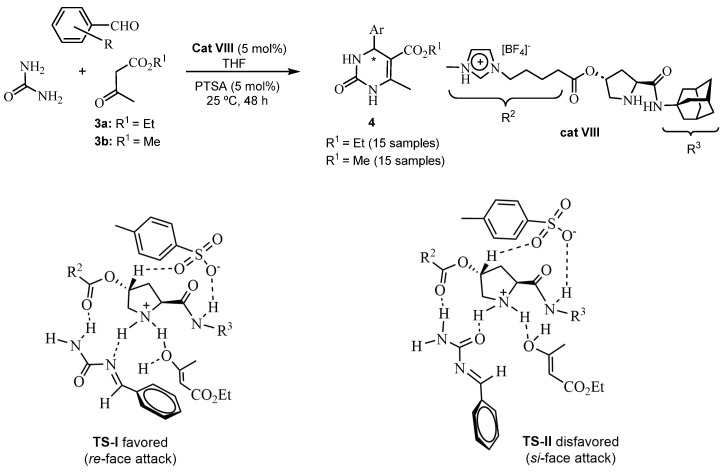
ABR with **cat VIII** and plausible transition state (**TS-I**) to favor re-face attack.

Deepa et al. in 2022 [44] also used imidazolium ionic liquids in an ABR, but with adamantyl (L)-prolinamide and [BF_4_]^−^ as the anion (**cat VIII**) and p-toluenesulfonic acid as an additive (see Scheme 11). A series of 30 chiral DHPMs were obtained in 16–74% yields and up to 85% *ee* (25 °C, 48 h). A feasible reaction mechanism and transition states or intermediates were proposed and supported by FT-NMR spectroscopy and DFT calculations. Thus, these studies showed a favored *re*-face attack on the imine bond, giving the (*R*)-configuration by asymmetric induction.

#### 2.1.3. Chiral Amines as Catalysts

**Scheme 12:**
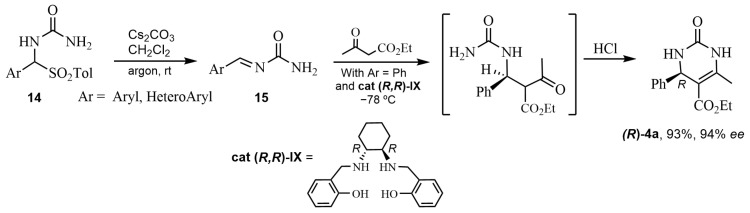
Enantioselective synthesis of DHPMs **(R)-4a** from alkylidene ureas with **cat (R,R)-IX**.

**Figure 3:**
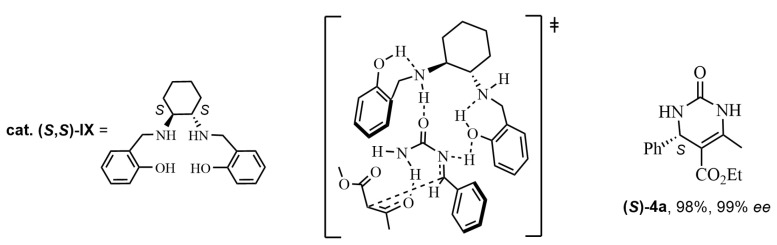
Transition state for **cat (*S,S*)-IX** by a network of cooperative hydrogen bonds (NCHB) in the synthesis of the DHPMs **(*S*)-4a**.

**Scheme 13:**
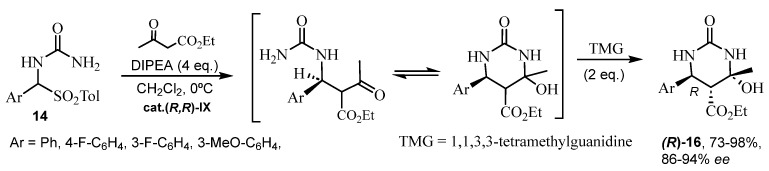
Synthesis of the HHPMs **16** by an ABR catalyzed by cat **(*R,R*)-IX**.

As a matter of fact, numerous chiral organocatalysts in ABRs are amine compounds. Already in the early 2010s, a chiral primary amine catalyst was used by Xu et al. [45]. On the other hand, amines derived from cinchona alkaloids were assessed as organocatalysts for ABRs by Ding and Zhao, who proposed a feasible transition state for the formation of the obtained (*R*)-enantiomer (up to 78% *ee*) [46]. In 2016, Saá’s research group [47,48] obtained DHPMs with excellent enantioselectivities by asymmetric reaction with chiral catalysts (**cat IX**). Considering the via-imine mechanism for the Biginelli reaction, ten arylideneureas **15** were previously synthesized and later condensed with ethyl acetoacetate. Taking into account the mechanisms of biocatalysts, these authors chose these arylideneureas **15**, which could act as donor-acceptors forming a network of cooperative hydrogen bonds (NCHB) with chiral amines (*R,R*)-I or (*S*,*S*)-I as organocatalysts (Scheme 12 and Figure 3). Acid treatment afforded the (*R*)-DHPMs **4a** in good yields and enantioselectivities (Scheme 12). Similarly, in the second publication [48], the direct combination of an α-ureidosulfone **14** with an alkyl β-ketoester in the presence of a suitable organic base and the organocatalyst was carried out. The treatment of the crude Mannich mixture with acid led to DHPMs (***S***)-**4a** (Figure 3), while organic base (TMG) treatment yielded hexahydropyrimidinones (HHPMs) **16** (Scheme 13). It is worth noting the formation of three chiral centers in these HHPMs **16**, which could be isolated without dehydration.

High enantioselectivity was achieved using a primary amine with an amide backbone as a chiral catalyst. Some authors studied the origin of the observed enantioselectivity by using density functional theory (DFT). The rate-determining step was the proton transfer process in the cyclization of the substrate, mediated by the amide moiety of the catalyst. These authors concluded that the orientation of the amide moiety was the determinant fact for the observed enantioselectivity [49].

**Figure 4:**
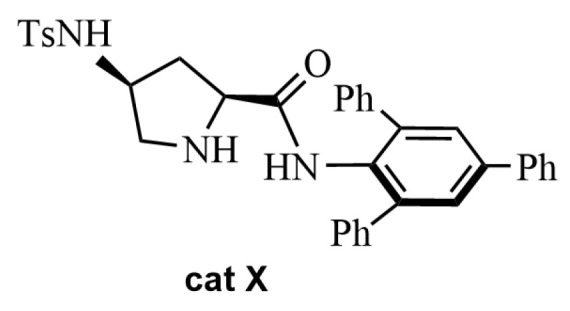
The catalyst **cat X** used by Saha and Moorthy in the ABR [51].

**Scheme 14:**
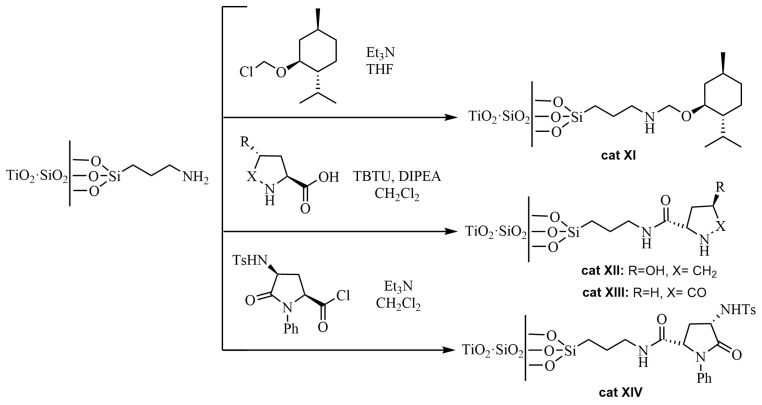
Synthesis of chirally modified TiO_2_·SiO_2_ nanoparticles containing (3-aminopropyl)triethoxysilane.

Proline derivatives and analogues also represent appropriate options to achieve enantiomerically pure products in asymmetric Biginelli reactions. In 2015, Titova et al. [50] continued the use of amides of proline previously reported by Saha and Moorthy, who built a sterically hindered catalyst in 2012 (Figure 4), which rendered high selectivities [51]. In addition to modified prolines, menthol derivatives were employed in the new experiments [50]. These chiral molecules were immobilized on the surface of mixed nanoparticles of titanium and silicon oxides (TiO_2_·SiO_2_) containing (3-aminopropyl)triethoxysilane. The mixtures produced idoneous catalysts to promote chiral adducts increasing the selectivity observed in the ABR (Scheme 14) [50].

**Scheme 15:**
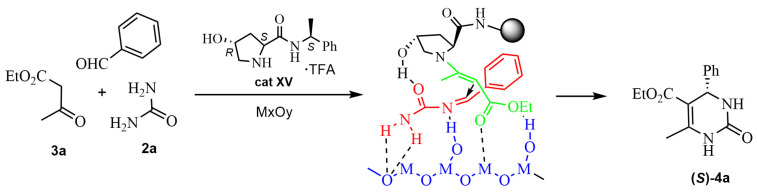
Asymmetric catalyzed reaction with chiral proline derivative and metal oxides.

New experiments were carried out by the same group [52] combining another chiral proline-TFA derivative organocatalyst **cat XV** with titanium dioxide or alumina as heterogeneous catalysts. These additives were incorporated singly or mixed, in bulk or in nanosized form. Variations over reaction conditions were studied in addition to the role of the components, catalysts, and additives. A plausible transition state of the asymmetric reaction was also proposed (see Scheme 15).

**Figure 5:**
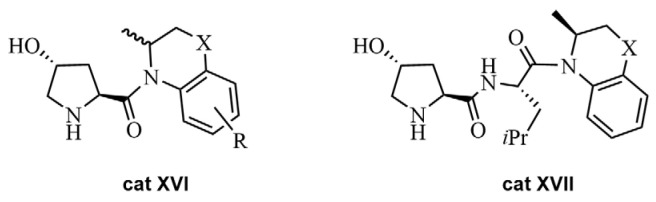
Prolinamides used alone or in combination with nanosized oxides in an ABR.

Prolinamides were found to be better catalysts in asymmetric organic reactions compared to proline in most cases [53]. Thus, prolinamide derivatives of 4-Hydroxy-(2S)-prolines bearing 1,2,3,4-tetrahydroquinoline or 3,4-dihydro-2*H*-1,4-benzoxazine or diverse substituents were synthesized and treated with nanosized oxides SiO_2_·ZrO_2_ to eventually use these combinations as heterogeneous chiral catalysts in an ABR [54]. An increase in the *ee* values of the reaction product was observed when nanoparticles of SiO_2_·ZrO_2_ were added to the chiral proline derivatives **cat XVI** or **cat XVII** (Figure 5), from 54% (without oxides) up to 76%.

**Figure 6:**
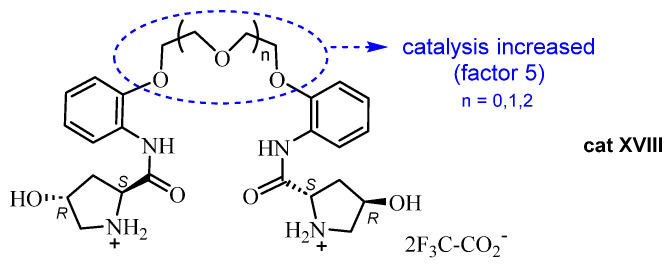
Prolinamides with a polyether spacer used as catalysts.

A variation of this methodology by these authors consisted of the incorporation of a polyether spacer in the (2*S*,4*R*)-hydroxyproline derivative **cat XVIII** [55], allowing the authors to enhance the effect on the enantioselectivity of the Biginelli reaction by a factor of five. The best results were achieved for salts of these podands with trifluoroacetic acid (Figure 6).

**Figure 7:**
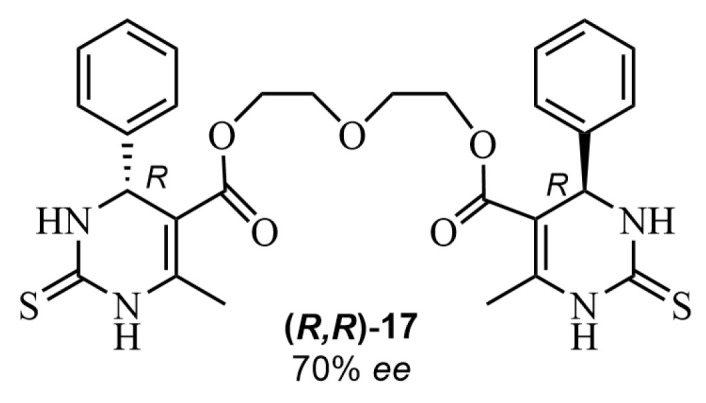
A dihydropyrimidinethione-containing podand (***R,R***)-**17** obtained by ABR.

**Figure 8:**
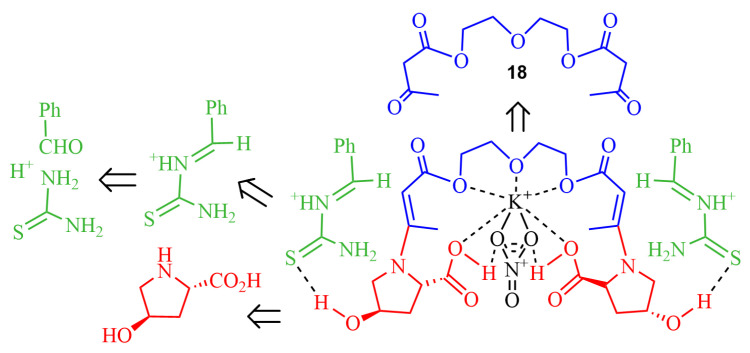
Complexation of the podand **18** with metal ion in the transition state of ABR with iminium ions and hydroxy prolines.

The methodology was extended by using a variety of nanosized metal oxides (NiO, Fe_3_O_4_, CuO, Al_2_O_3_, SiO_2_, and TiO_2_·SiO_2_) [56]. The dihydropyrimidinethione-containing podand (***R,R***)-**17** (Figure 7) was synthesized by an asymmetric Biginelli-like reaction. The best result was achieved in the presence of nanooxide of NiO and 4-hydroxy-L-proline under mild conditions (70% *ee* and a moderate yield). Related experiments reported by these authors encompassed metal salts (especially metal nitrates) as additives, to study plausible complexes’ formation in the transition states (Figure 8) [57]. In another publication, it was shown that the combined system L-proline/SiO_2_·Mg(OH)_2_ as a catalyst led to an enhanced enantiomeric excess of some ABR products of up to 18% [58]. The *ee* values were determined by high-efficiency liquid chromatography.

With the aim of testing the effects of chiral inductors based on 4-hydroxy-L-proline podands (Figure 6, n = 2) on the selectivity of ABR, a theoretical study was performed [59]. The results showed that when the podand was in a base form, the interaction of a podand–acetoacetate adduct with N-benzylideneurea was preferred, while for a salt form, the interaction with benzaldehyde was more favorable (via iminium), which led to a higher stereoselectivity.

**Scheme 16:**
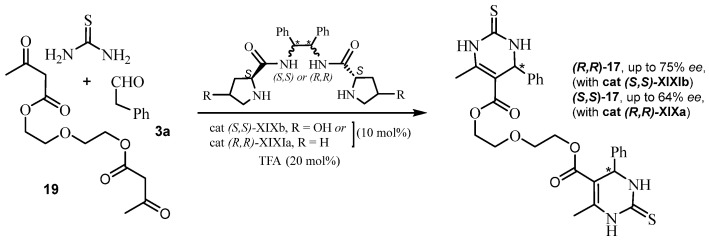
Synthesis of the podand DHPTs **(*R,R*)-17** and **(*S,S*)-17**.

Very recently, a new work has been published by this group [60]. This work showed the relevant role of the hydroxyl group of the chiral inductor **cat (*S,S*)-XIXb** (based on *trans*-4-hydroxyproline) in obtaining the target podand **17**, enriched with the (*R,R*)-enantiomer. Considering the iminium mechanism, the hydroxyl group promotes the prochiral *Si*-face of the Schiff base with a maximum of 75% *ee* (Scheme 16). It should be noted that a known hydroxyphenyl analogue of **17** inhibits the activity of the motor protein Eg5.

#### 2.1.4. Other Chiral Organic Catalysts

**Scheme 17:**
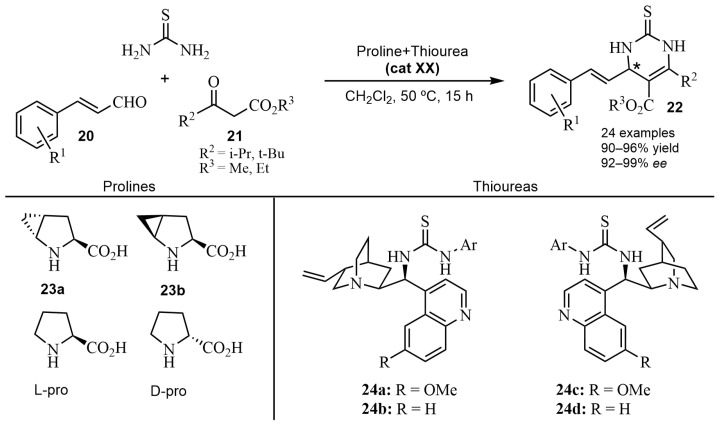
Synthesis of the DHPTs **22** with the combined **cat XX** of proline/thiourea.

**Scheme 18:**
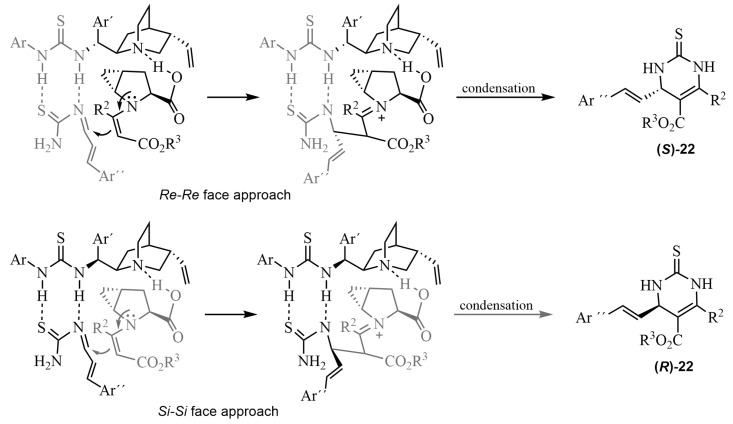
Plausible reaction mechanism for an ABR with the combined **cat XX** of proline/thiourea.

A number of asymmetric multicomponent reactions are catalyzed by thiourea-based organocatalysts. Thus, in ABRs, chiral organic compounds possessing a thiourea skeleton have been shown to be useful in the enantioselective formation of dihydropyrimidine thiones [61]. Early work was reported with a bifunctional primary amine, with TfOH as a chiral phase-transfer catalyst, and with t-BuNH_2_·TFA as an additive, showing moderate enantioselectivities [62]. Good examples are the syntheses depicted in Scheme 17. A series of α,β-unsaturated aldehydes **20** and various β-ketoesters **21** reacted in an ABR catalyzed by a proline/thiourea-based dual system (**cat XX**) [63,64]. Preliminary studies [63] were carried out with methyl or ethyl isobutyrylacetate in various solvents and different catalyst mixtures of prolines and thioureas, to find the optimum reaction conditions. The best results for the DHPTs **22** were obtained with the proline **23a** (5 mol%)/thiourea **24a** (5 mol%), providing excellent yields (90–96%) and high enantioselectivities (92–99% *ee*) [64]. A plausible transition state was proposed to explain the observed asymmetric induction. The role of the H-bonding, involved in the process, appeared to be crucial in the formation of the transition state (Scheme 18).

##### Organocatalyst with Metallic Cations

**Scheme 19:**
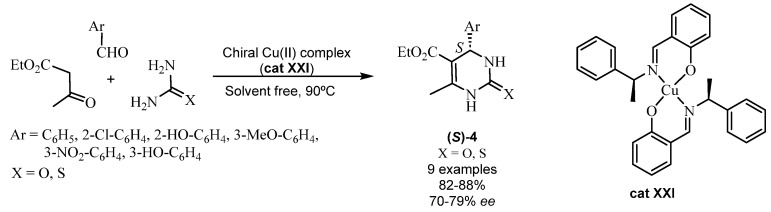
Synthesis of the DHPTs **21** with a chiral Cu (II) complex (**cat XXI**).

A variety of organocatalysts can form complexes with metallic cations in Biginelli reactions. Metallic complexes have been employed for some time in MCRs. In 2015, Kamali reported [65] an enantioselective synthesis of the dihydropyrimidines **4** using the chiral Schiff base copper (II) complex **cat XXI** as a chiral catalyst. The participation of **cat XXI** in an ABR allowed the obtainment of the (*S*) enantiomers of 3,4-dihydropyrimidine-2-ones **(*S*)-4** (X=O) and their sulfur analogs 3,4-dihydropyrimidine-2-thiones **(*S*)-4** (X=S) (Scheme 19).

**Scheme 20:**
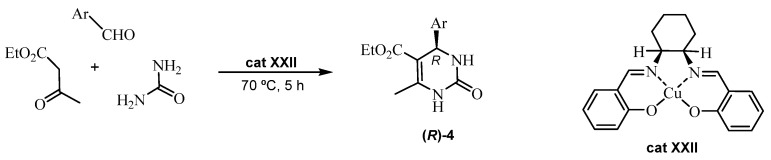
ABR catalyzed by an encapsulated chiral Cu(II) salen catalyst (**cat XXII**).

Another Cu(II)–salen complex (**cat XXII**) encapsulated in MWW-zeolite framework pores showed its efficiency as an chiral organocatalyst in the enantioselective formation of the DHPMs derivatives **(*R*)-4** (Scheme 20) [66]. The structural properties of **cat XXII** were confirmed by spectroscopic methods. In addition, computer-assisted DFT calculations were essential to study the process (Scheme 20).

##### Boranes

**Scheme 21:**
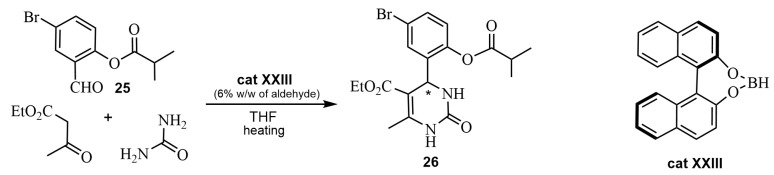
Synthesis of the DHPM **26** with **cat XXIII**.

Chiral binol borane has been used as a Lewis acid catalyst in ABRs. For instance, the reaction of the 5-Bromo-2-acyl salicylaldehyde **25** with ethyl acetoacetate and urea using **cat XXIII** in THF led to a bromopyrimidine **26** [67]. The absolute configuration of a single crystal **(*S*)-26** was determined by X-ray (Scheme 21).

#### 2.1.5. Biocatalysts

**Scheme 22:**
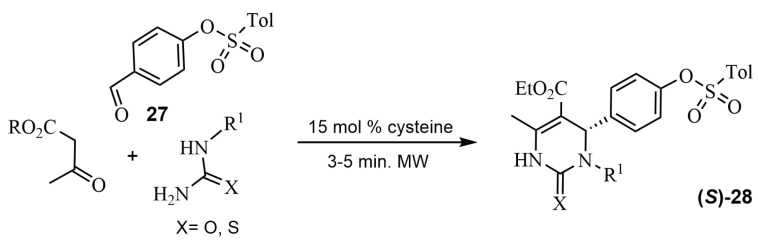
Synthesis of the DHPM **28** in the presence of cysteine.

Several ABRs using biocatalysts have been described in the last few years. For example, in 2013, bovine serum albumin (BSA) was chosen as a natural catalyst, allowing for a gram-scale synthesis of Monastrol, even though selectivities were not good [68]. Later, in 2022, Elkanzi et al. [69] carried out a Biginelli reaction in the presence of cysteine as a new green bio-organic catalyst. Although the catalyst was chiral, the authors did not provide any data on enantioselectivity. Nevertheless, they performed docking studies on certain enantiomers such as **(*S*)-28** (Scheme 22). All compounds were studied by molecular docking and subsequently their antifungal activities were evaluated.

**Scheme 23:**
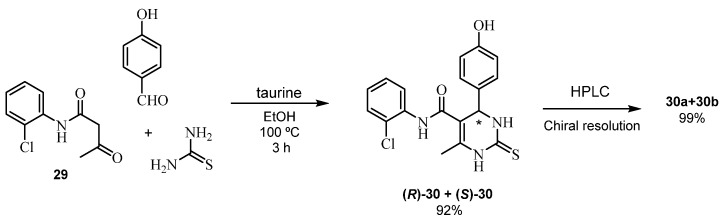
Synthesis of racemic **(*R,S*)-30** in the presence of taurine and their chiral resolution by HPLC.

In a recent work [70], taurine showed its efficiency as a green and reusable bio-organic catalyst (up to 99% yield). In fact, this is not an example of asymmetric reaction, but we highlight it as a current example of efficient enantiomer separation. Chiral resolution of the twenty-three derivatives of the (R/S)-THPM-5-carboxanilides **30** was carried out by chiral HPLC (up to 99.99% purity). The absolute configuration of all the enantiomerically pure isomers was determined using a circular dichroism study and validated by a computational approach (Scheme 23).

#### 2.1.6. Chiral Starting Compounds

**Scheme 24:**
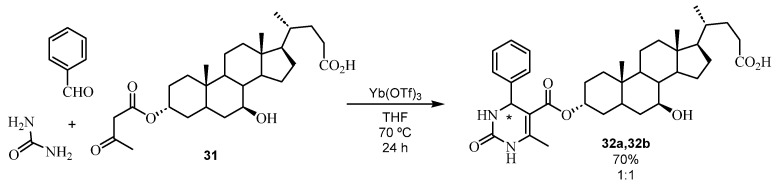
Synthesis of ursodeoxycholic-derived **32a, 32b**.

In addition to using chiral catalysts to achieve ABRs with good stereoselectivity, it is possible to perform asymmetric induction starting from chiral compounds. In the last few years, research studies have been carried out on formyl monosaccharide derivatives to achieve optically active DHPMs [71]. One single stereoisomer with the (*R*)-configuration was isolated. Recently [72], a novel Biginelli reaction utilized as an acetylacetate derivative an ester of ursodeoxycholic acid (UDCA), which is present in the human intestine and with the possible application that their hybrids could be potential therapeutic agents. Surprisingly, the chiral skeleton of the ester **31** did not perform sufficient chiral induction to selectively obtain one of the isomers. The diastereomeric DHPMs **32** were obtained in a 1:1 ratio with 70% yield (Scheme 24).

**Scheme 25:**
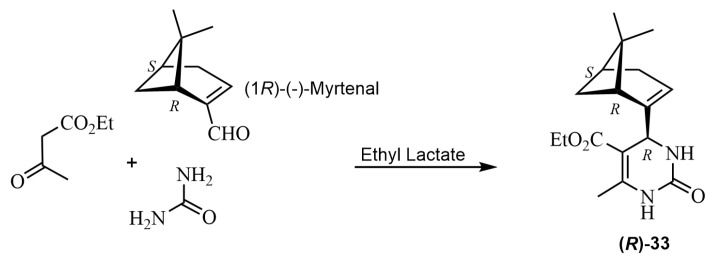
Synthesis of myrtenal derivative **(*R*)-33**.

Myrtenal was used as a chiral aldehydic substrate to produce a valuable asymmetric induction in the synthesis of THPMs [73]. To a mixture of ethyl acetoacetate and urea in ethyl lactate as a green solvent, (1*R*)-(−)-myrtenal was added. The result, after heating, was the formation of the isomer (**4*R***)**-33** as a white solid (Scheme 25). The authors highlighted the role of the chiral green solvent in combination with myrtenal to achieve the optimal diastereoselectivity. The product was characterized by several spectroscopic methods, and its structure was determined by X-ray crystallography.

#### 2.1.7. Nanoparticles as Catalyst Support

A number of works on Biginelli-catalyzed reactions in the presence of NPs have already been referenced [39,50,54,56]. In 2019, Jafari-Chermahini et al. reported the preparation of functionalized NPs from magnetite by successive application of blended natural polymers (gelatin and λ–carrageenan) on an Fe_3_O_4_ core [74]. It was studied the role of Gelatin as enantiomeric inductor in the catalytic active sites. On the other side, λ–carrageenan provides stability for gelatin immobilization on Fe_3_O_4_ nanoparticles. The amino acid sites, presented at the surface of the NPs, showed their efficiency as organocatalysts for ABRs. The corresponding DHPMs were obtained in good to excellent yields with 63–98% *ee*.

#### 2.1.8. Biginelli Related Reactions

##### Spirofuranes

**Scheme 26:**
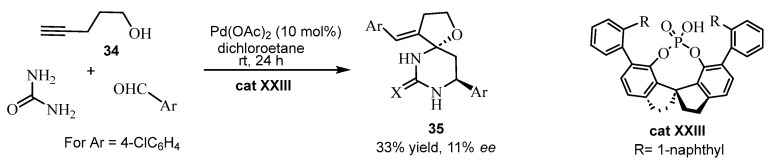
Synthesis of the spirofuran-hydropyrimidinone or -hydropyrimidinethione compounds **35**.

A variety of spirofuran-hydropyrimidinone or -hydropyrimidinethione compounds **35** were prepared from the alkynols **34** [75]. These compounds served as enolizable carbonyl equivalents to react with urea or thiourea and aromatic aldehydes, with co-catalysis of palladium chloride and trifluoroacetic acid, giving good yields and *trans* stereoselectivity An asymmetric version was carried out for Ar = *p*-chlorophenyl, with catalysis of Pd(OAc)_2_ and the chiral phosphoric acid **cat XXIII**, but **35** was obtained in only 33% yield and 11% *ee* (Scheme 26). In this case the results were not good, but the compound **35** could be useful given that it exhibits two asymmetric carbons.

##### Thiadiazine Derivatives

**Scheme 27:**
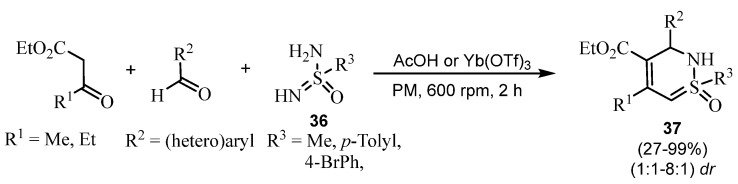
Synthesis of the 2,3-dihydro-1,2,6-thiadiazine 1-oxides **37** from the sulfonimidamides **36**.

Although the following reaction is not actually a synthesis of THPMs, we highlight it as being related to the Biginelli reaction and its potential as an asymmetric reaction. Krauskopf et al. [76] valued the stereogenic sulphur in sulfonimidamides to obtain the diastereoisomers **37** with variable selectivities. For example, the NH-free sulfonimidamides **36** provided the 2,3-dihydro-1,2,6-thiadiazine 1-oxides **37** in high yields by using acetic acid or ytterbium triflate as catalysts. The couplings were performed in a planetary ball mill (PM) under solvent-free mechanochemical conditions. The structure of one of the products was characterized by X-ray single crystal structure analysis (Scheme 27).

## 3. Conclusions

Over the last few years, there has been an important increase in the publication of asymmetric methods. There is a growing awareness of the need to stereoselectively obtain the correct isomer, especially in applications for pharmaceuticals. Unfortunately, some authors do not provide enough information about the configuration obtained when they carry out a potential ABR. Among the catalysts described in asymmetric syntheses, the organocatalysts have proven more useful. Research has been carried out not only in the search for new Brønsted catalysts, but also for chiral amine and chiral amide derivatives as well. Ionic liquids have also been shown to be useful in combination with chiral molecules. On the other hand, in addition to the use of chiral catalysts, the use of chiral substrates to perform internal asymmetric induction should also be highlighted (unfortunately with few examples in this field). Some naturally occurring molecules such as monosaccharides, amino acids, and other chiral derivatives should be studied as starting materials, particularly in the case of monosaccharides (with scarce recent literature on ABRs), whose structures can be modified in numerous ways for eventually improving asymmetric induction.

## Data Availability

No data are available.

## Acronyms and Abbreviations

ABR	Asymmetric Biginelli reaction
ACDC	Asymmetric counteranion-directed catalysis
ILE	Ionic liquid effect
BINOL	1,1′-bi-2-naphthol
DHPMs	Dihydropyrimidinones
DHPMT	Dihydropyrimidinethiones
DIPEA	Diisopropylethylamine
DMF	Dimethylformamide
dr	Diastereomer relation
*ee*	Enantiomeric excess
HHPM	Hexahydropyrimidinones
MCRs	Multicomponent reactions
MS	Mass spectroscopy
MSI	1-Methyl-3-(3-sulfopropyl)-1*H*-imidazol-3-ium
NCHB	Network of cooperative hydrogen bonds
NP	Nanoparticle
MW	Microwave
PM	Planetary ball mill
PTSA	*p*-Toluenesulfonic acid
Pro	Proline
rt	Room temperature
ref	Reference
US	Ultrasound
TBTU	2-(1*H*-Benzotriazole-1-yl)-1,1,3,3-tetramethylaminium tetrafluoroborate
THF	Tetrahydrofuran
THPMs	Tetrahydropyrimidines
TMG	1,1,3,3-Tetramethylguanidine
TFA	Trifluoroacetic acid
TMSCl	Trimethylsilane chloride
TRIP	(*R*)-3,3′-Bis-(2,4,6-triisopropylphenyl)-1,1′-binaphthyl-2,2′-diyl phosphate
